# Tuberous sclerosis complex: A clinical diagnosis in Ethiopian patients

**DOI:** 10.1097/MD.0000000000037135

**Published:** 2024-02-09

**Authors:** Belete Sisay, Abilo Tadesse, Abebe Gelaw, Desalew Getahun, Biruk Mulat, Weynishet Kebede, Yonathan Gebrewold

**Affiliations:** a Department of Internal Medicine, College of Medicine and Health Sciences, University of Gondar, Gondar, Ethiopia; b Department of Radiology, College of Medicine and Health Sciences, University of Gondar, Gondar, Ethiopia.

**Keywords:** inherited disorder, Northwest Ethiopia, tuberous sclerosis complex

## Abstract

**Rationale:**

Tuberous sclerosis complex (TSC) is a rare autosomal dominant inherited disorder characterized by the development of nonmalignant tissue growths (hamartomas) in various organ systems, often located in the brain, skin, heart, lung and kidneys. The delayed diagnosis could be attributed to low expectation or exposure of physicians to this rare disease. High index of clinical suspicion is required for early diagnosis of rare diseases to prevent adverse outcomes.

**Patient concerns:**

The first patient, a 27-year-old man, presented with intermittent left flank pain and hematuria of 5 months duration. On examination of the skin and oral cavity, he had fibrous cephalic plaque, facial angiofibromas, ungual fibromas, confetti skin lesions, and intraoral fibromas. A CT scan of the chest, abdomen, and brain displayed cystic lung parenchymal changes and multifocal micronodular pneumocyte hyperplasia, angiomyolipomas in both kidneys, and multiple calcified subependymal nodules (SEN), respectively. The second patient, a 28-year-old woman, presented with a seizure disorder in the last 1 year, and papular and nodular lesions over her face since childhood. On examination of the skin and oral cavity, she had hypomelanotic macules, facial angiofibromas, shagreen patches, ungual fibromas, intraoral fibromas, and dental enamel pits.

**Diagnoses:**

Definitive diagnosis of TSC was made in both patients using the “2012 tuberous sclerosis complex diagnostic criteria consensus statement.”

**Interventions:**

The first patient was seen by various medical discipline teams, and suggested close follow-up in the “chronic illness clinic” of the hospital. The second patient was scheduled in dermatology clinic for electrocautery for disfiguring facial nodules.

**Outcome:**

Both patients were scheduled for close follow-up in the hospital.

**Lessons:**

The patients described had TSC using “clinical diagnostic criteria.” Under the clinical diagnostic criteria of TSC, 4 of 11 major criteria and 3 of 7 minor criteria are skin features. Hence, awareness on skin features as clinical markers to suspect TSC should be emphasized in resource-limited countries.

## 1. Introduction

Tuberous sclerosis complex (TSC) is a rare autosomal dominant inherited disorder characterized by occurrence of nonmalignant tumors in various organ systems. It predominantly involves the brain, skin, heart, lung and kidneys.^[1–3]^ Detail on clinical description of TSC was first reported by Bourneville in 1880.^[4]^ The annual incidence of TSC is 1 case per 6000 to 10,000 live births. TSC affects all age groups and races with no gender predilection.^[1–3]^ It is caused by pathogenic mutation of tumor suppressor gene, either TSC1 or TSC2, which regulates mechanistic target of rapamycin complex-1 (mTORC1) transduction signaling pathway. Deregulated activation of mTORC1 results in development of benign tumors in various organ systems due to unchecked cellular hyperplasia.^[1–3,5–7]^ Diagnosis of TSC is made by the “2012 TSC diagnostic criteria consensus statement,” which consists of “clinical diagnostic criteria” by using clinical signs and radiologic findings, and “genetic diagnostic criteria” by detecting pathogenic TSC genes mutation.^[8,9]^ Here, we present 2 cases of TSC diagnosed by clinical diagnostic criteria.

## 2. Case presentation

### 2.1. Case-1

**Figure 1:**
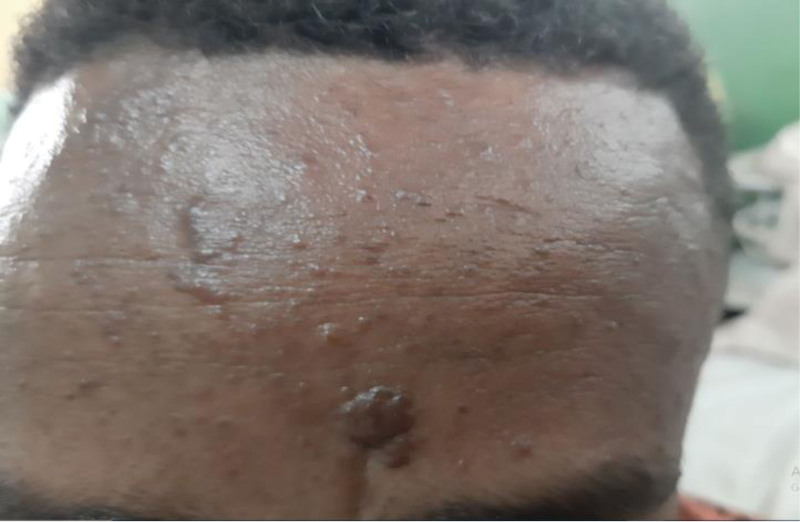
Fibrous cephalic plaque (case-1).

**Figure 2:**
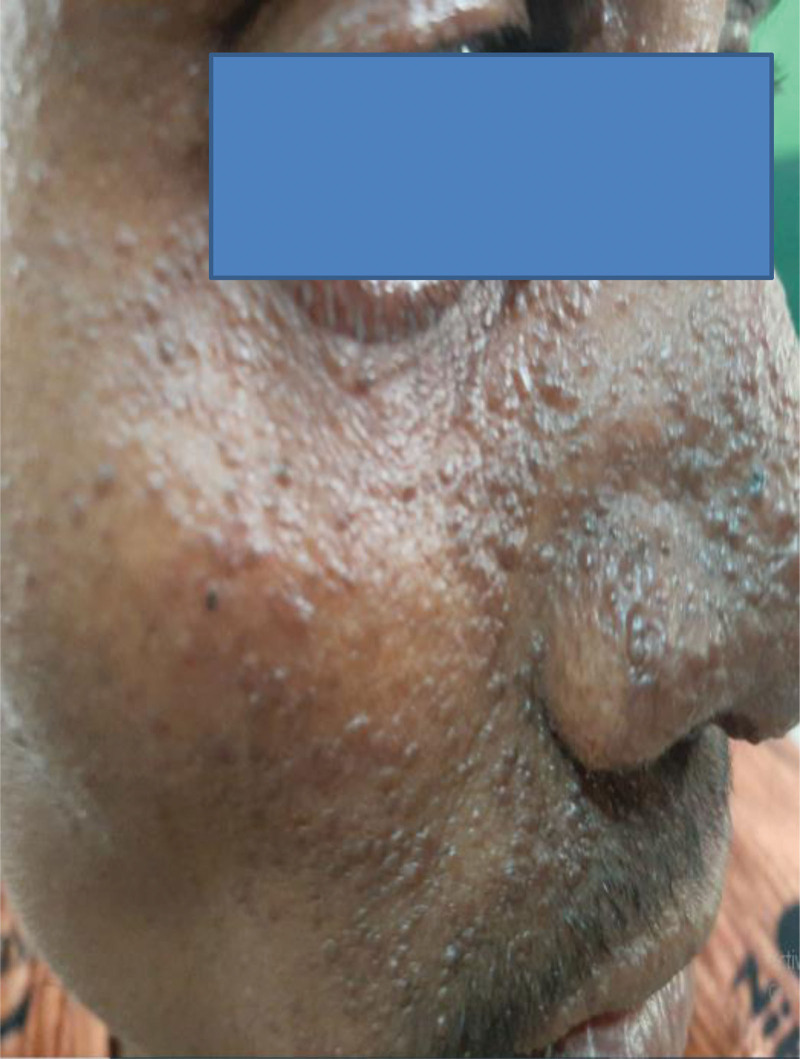
Facial angiofibromas (case-1).

**Figure 3:**
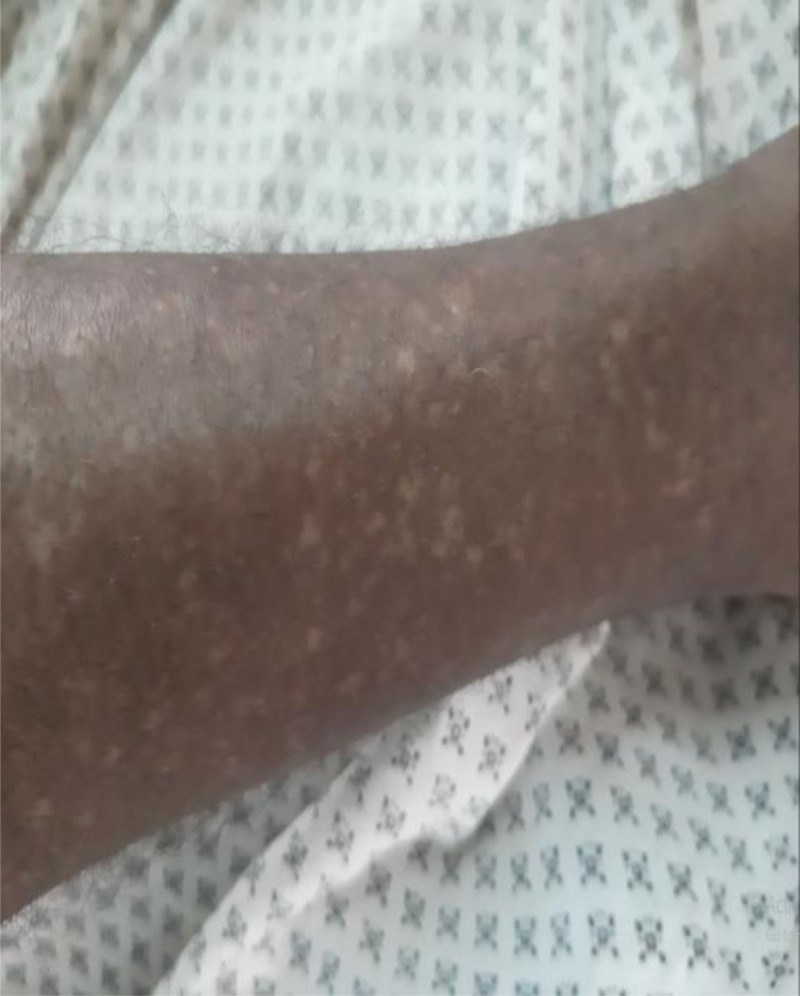
Confetti lesions (case-1).

**Figure 4:**
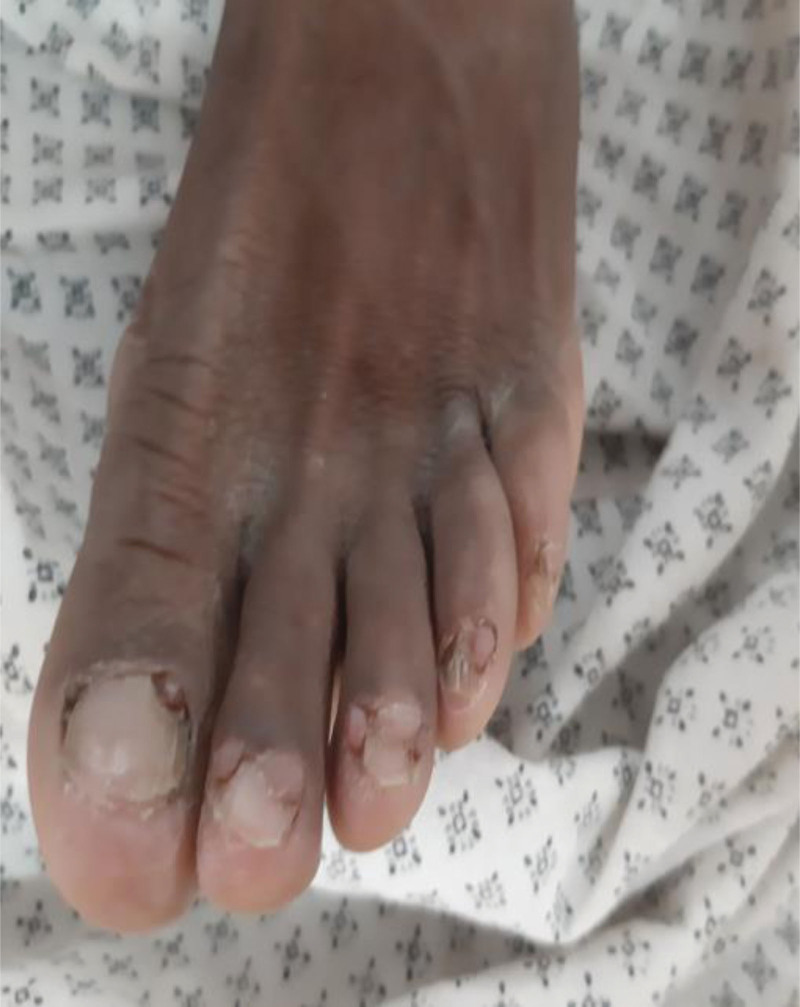
Ungual fibromas (case-1).

**Figure 5:**
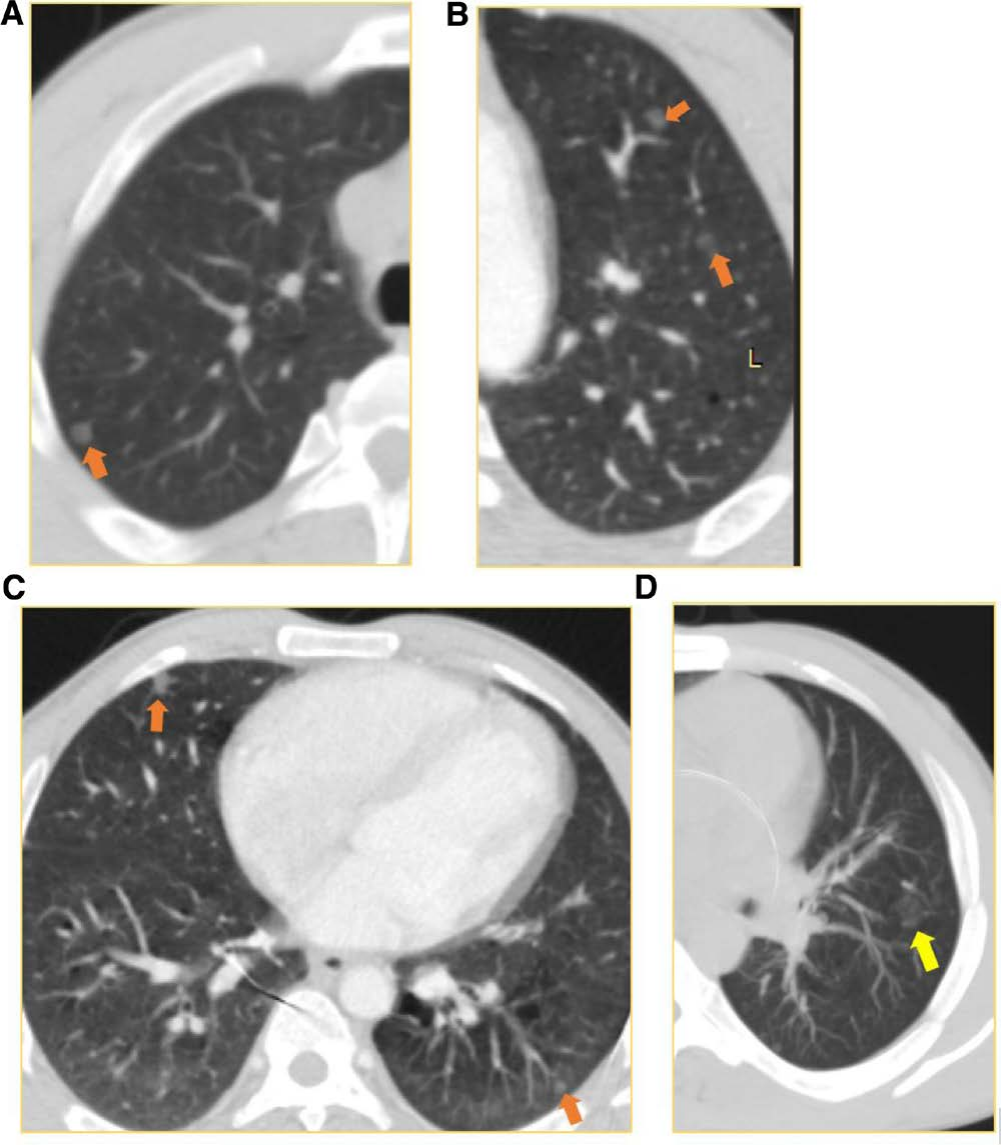
HRCT of the Lung (case-1): Multiple Small (≤ 5mm) well defined randomly distributed ground glass nodules in both lungs (orange arrows in A–C and yellow arrow in D). The characteristics and distribution of the nodules is suggestive of multifocal micronodular pneumocyte hyperplasia (MMPH). There are cystic parenchymal changes in both lungs (B, C).

**Figure 6:**
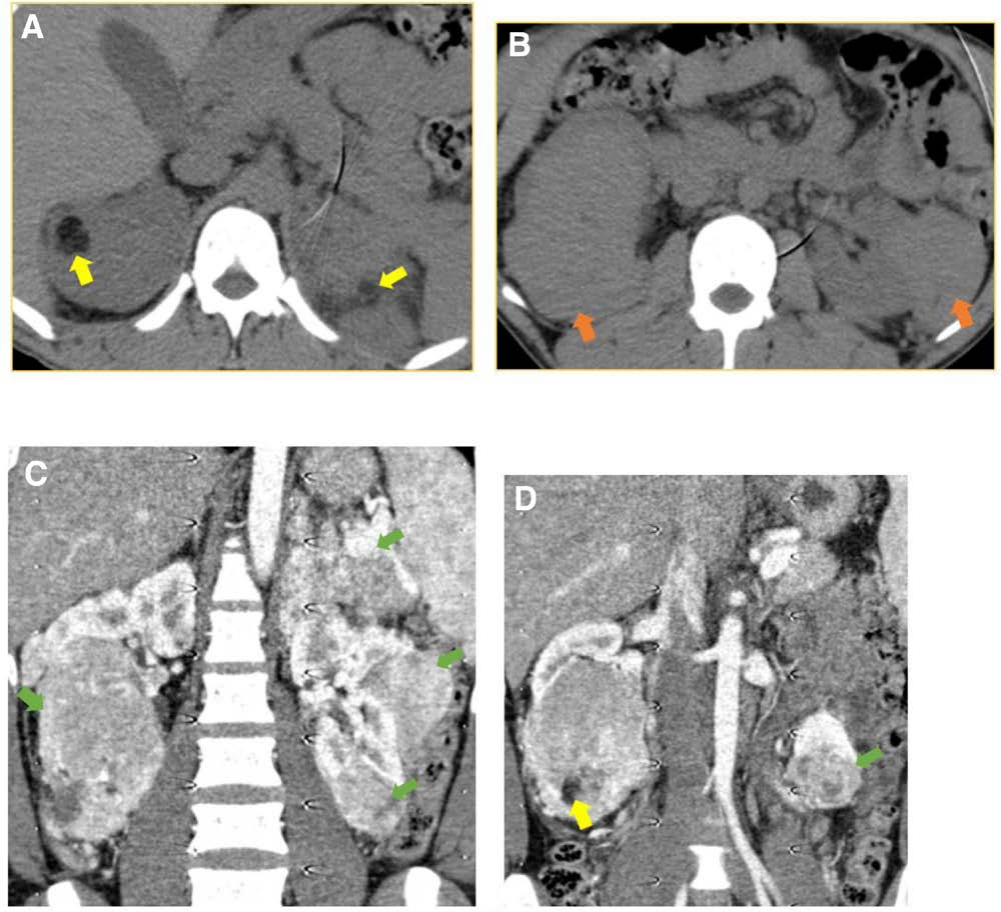
Abdominal CT scan (case-1): The pre contrast study reveals slightly hyperdense, well defined, mildly heterogeneous fat containing (yellow arrows) masses in both kidneys. The masses have variable sizes with ball type, exophytic growth pattern. The post contrast study reveals mildly heterogeneous enhancement (green arrows). Multiple vessels are also seen coursing through the masses (C, D). The findings are consistent with multiple angiomyolipomas (AML) in both kidneys.

**Figure 7:**
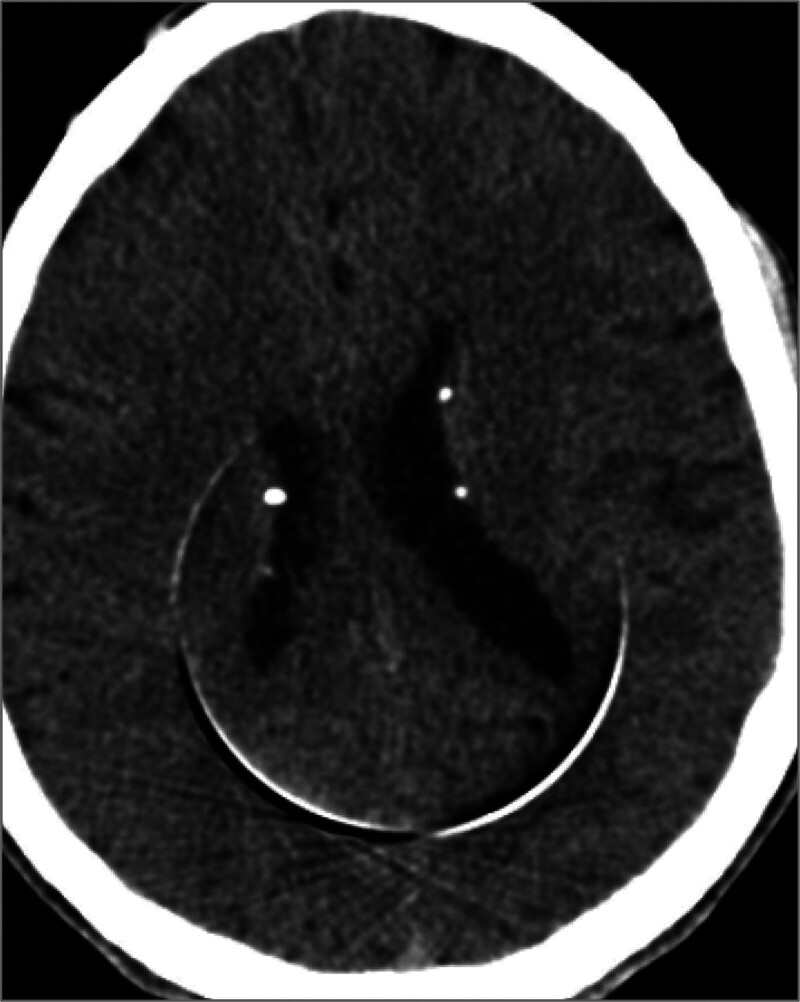
Plain CT scan of the brain (case-1): Multiple calcified subependymal nodules (hamartomas) of tuberous sclerosis.

A 27-year-old man presented with the complaint of intermittent left flank pain and hematuria of 5 months duration. He developed progressively worsening papular skin lesions over his face in the past 4 years, which later involved his neck and upper back. He noticed multiple hypopigmented skin lesions over his legs and nodular lesions over the nail beds of fingers and toes in the last 2 years. He had intermittent global headache, which was responsive to available oral analgesics. He had no abnormal body movement, blurred vision, or weakness of extremities. He had no cough, shortness of breath or chest pain. He had no orthopnea, palpitation or leg swelling. There was no similar illness in the family. On physical examination, vital signs were within normal limits. Pertinent findings were on integumentary and genitourinary system. He had 1.5 cm × 2 cm skin plaque over his forehead (fibrous cephalic plaque). He had several papular and nodular lesions over his nose and cheeks distributed in a butterfly pattern (facial angiofibromas), multiple nodules over the nail beds of fingers and toes (ungual fibromas), multiple nodular lesions on gingivae (intraoral fibromas), and several hypopigmented spots over his legs (confetti skin lesions) (Figs. 1–4). There was bimanually palpable, firm in consistency, smooth surfaced, tender mass on the left flank region, which was likely to be enlarged left kidney. There was left costo-vertebral angle tenderness. Findings on the respiratory, cardiovascular, and nervous system examinations were unremarkable. No abnormality was seen on fundoscopic examination. On laboratory evaluation, hemogram revealed hematocrit (HCT) = 35%, hemoglobin = 11.7 gm/dL. White blood cell and platelet counts were within normal range. Urinalysis revealed blood 3 + and protein trace to 1 + on deep stick, but no cells or urinary casts. Serum biochemical tests revealed serum creatinine (Cr) = 1.02 mg/dL. Serum liver biochemical tests and electrolytes were within normal limits. On imaging evaluation, CT scan of the chest revealed cystic lung parenchymal changes and multiple Small (≤5 mm) well-defined randomly distributed ground glass nodules in both lungs (multifocal micronodular pneumocyte hyperplasia). Abdominal CT scan showed well-defined, heterogeneously enhancing, fat and vascular structure containing, exophytic bilateral renal masses (renal angiomyolipomas). CT of the brain displayed multiple calcified subependymal nodules (SEN) (Figs. 5–7). Echocardiography and ECG revealed normal findings. Clinical diagnosis of TSC was made based on the “2012 TSC diagnostic criteria consensus statement,” since the patient was found to have 4 major clinical criteria (≥3 facial angiofibromas)/ fibrous cephalic plaque, ≥2 ungual fibromas, SEN, and ≥ 2 renal angiomyolipomas), and 2 minor clinical criteria (confetti skin lesions and ≥ 2 intraoral fibromas). He was seen by various medical discipline teams, and suggested close follow-up in the “chronic illness clinic” of the hospital.

### 2.2. Case-2

**Figure 8:**
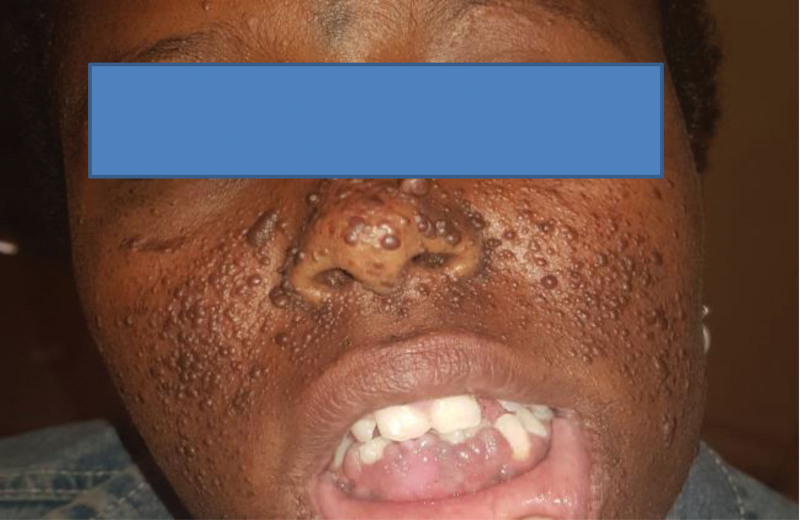
Facial angiofibroma (case-2).

**Figure 9:**
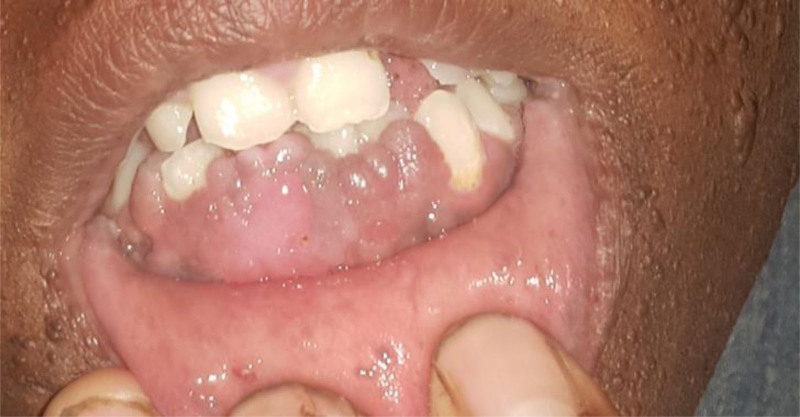
Gingival fibroma (case-2).

**Figure 10:**
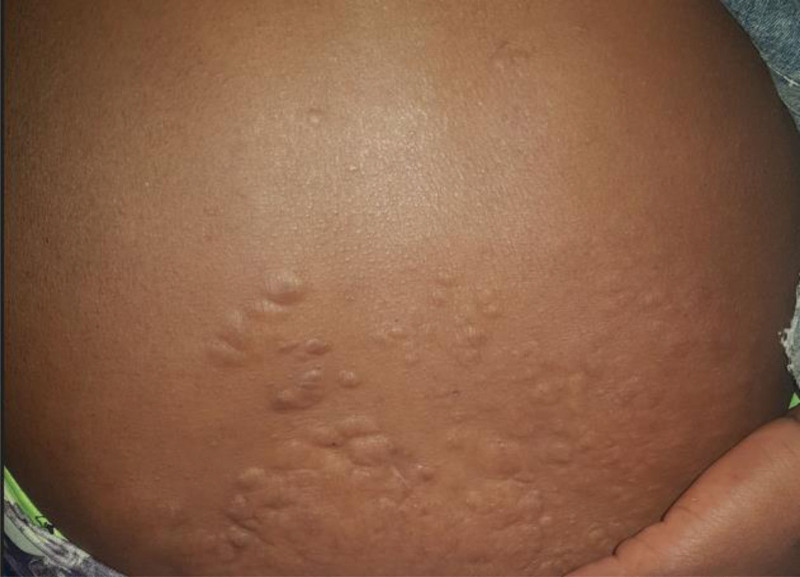
Shagreen patches (case-2).

A 28-year-old woman presented with the complaint of gradually progressive papular and nodular skin lesions over her face, which had been noticed since childhood. She was diagnosed with epilepsy 1 year ago in a primary care hospital and started on phenobarbital 100mg po twice daily, which she tolerated well. She had no headache, memory deficit, blurred vision, or extremity weakness. She had no cough, shortness of breath, or chest pain. She had no history of orthopnea, palpitation or leg swelling. She had no flank pain or hematuria. There was no similar illness in the family. On physical examination, vital signs were within normal range. Pertinent findings were on integumentary system. She had multiple papular and nodular lesions on her face with prominence on her nose and cheeks distributed in a butterfly pattern (facial angiofibromas), multiple gingival nodules (intraoral fibroma) and dental enamel pits, nodular lesions over the nail beds of fingers and toes (ungual fibromas), confluent patchy skin lesions over her lower back (shagreen patches), and multiple hypopigmented macular lesions over her thigh and legs (hypomelanotic macules) (Figs. 8–10). Findings on the respiratory, cardiovascular and nervous system examinations were unremarkable. Fundoscopic examination was non-revealing. On laboratory evaluation, hemogram, serum biochemical tests including renal function test, liver function tests and serum electrolytes were within normal range. There were no abnormalities on chest X-ray and ultrasound of the abdomen. Brain imaging was not done due to logistic reasons. Clinical diagnosis of TSC was made based on the “2012 TSC diagnostic criteria consensus statement,” since the patient was found to have 4 major clinical criteria (≥3 hypomelanotic macules, ≥2 facial angiofibromas, ≥2 ungual fibromas, and shagreen patches), and 2 minor clinical criteria (≥2 intraoral fibromas and ≥ 2 dental enamel pits). She was scheduled in dermatology clinic for electro cautery for disfiguring facial nodules.

## 3. Discussion

TSC is an inherited neurocutaneous disorder, named as phacomatosis. It is caused by loss-of-function mutation of tumor suppressor gene, either TSC1 or TSC2, which results in deregulated constitutive activation of mechanistic target of rapamycin complex-1 (mTORC1) transduction signaling pathway. Deregulated activation of mTORC1 causes unchecked cellular growth and proliferation, and results in nonmalignant tissue growth (hamartoma) in various organ systems.^[1–3]^ Two-thirds of cases of TSC are after de novo mutations, while one-third is inherited. Pathogenic variant TSC1 and TSC2 genes have variable penetrance, and result in variable presentations at any age group and even within affected family members.^[1–3,5–7]^ The main clinical presentations are neurologic (90%) with epilepsy, cortical dysplasia, and TSC-associated neuropsychiatric disorders (TAND); dermatologic (75–90%) with hypomelanotic macule and facial angiofibroma; renal (75–80%) with angiomyolipoma; cardiac (65–75%) with cardiac rhabdomyoma; ocular (50%) with intraretinal hamartoma; and pulmonary (35–50%) with lymphangioleiomyomatosis.^[1–3,8–10]^ Diagnostic suspicion of TSC begins at prenatal detection of cardiac rhabdomyoma and cerebral cortical tuber. Other clinical features are detected at distinct developmental points of postnatal life, such as hypomelanotic macule and retinal hamartoma during infancy to childhood; facial angiofibroma, SEN and renal angiomyolipoma during childhood to adulthood; and pulmonary lymphangioleiomyomatosis during adolescence to adulthood.^[1–3,8–10]^ Diagnosis of TSC is established by the “2012 international TSC diagnostic criteria consensus statement,” which consists of clinical and genetic diagnostic criteria. Clinical diagnosis of TSC is made by 2 major criteria, or 1 major and 2 or more minor criteria for definitive diagnosis. Possible diagnosis is made by 1 major only, or 2 or more minor criteria. Genetic diagnostic criteria require detection of pathogenic TSC1 or TSC2 gene mutations from normal tissues.^[8,9]^ Diagnoses of TSC in our patients were made by using “clinical diagnostic criteria,” since genetic testing was not available in the setting (Table 1). The main causes of morbidity or mortality associated with TSC are intractable epilepsy from cortical dysplasia, obstructive hydrocephalus from subependymal giant cell astrocytoma (SEGA), renal failure or hemorrhage from renal angiomyolipoma (AML), and recurrent pneumothoraces or respiratory failure from pulmonary lymphangioleiomyomatosis (LAM).^[1–3,8–10]^ Even though therapy for TSC is largely symptomatic, long lasting targeted therapy with cytostatic mTOR inhibitors, such everolimus and sirolimus have shown regression in size of brain tumor (SEGA) not amenable for resection, renal AML > 3 cm, and pulmonary LAM with rapid progression or moderate to severe lung disease. Better seizure control and delayed decline in lung function were reported with mTOR inhibitors use in patients with symptomatic cortical tuber and pulmonary LAM, respectively.^[7–11]^ Diseases of genetic origin are given less emphasis in sub-Saharan Africa and managed as low-priority cases as overburdened by infectious diseases. Under the clinical diagnostic criteria of TSC, 4 of eleven major criteria and 3 of 7 minor criteria are skin features. Hence, awareness on skin features as clinical markers to suspect TSC should be emphasized in resource-limited countries.^[12,13]^ Implementing the “2012 TSC diagnosis, surveillance and management recommendations” in sub-Saharan Africa would be challenging due to lack of skilled medical expertise, limited access to imaging modalities, unavailability of genetic testing, inaccessible targeted therapies (mTORi), and scarcity of resources.^[12,13]^

**Table 1:** 2012 updated diagnostic criteria for tuberous sclerosis complex.^[8,9]^

A. Genetic diagnostic criteria
The identification of either a TSC1 or TSC2 pathogenic mutation in DNA from normal tissue
B. Clinical diagnostic criteria
Major features
1. Hypomelanotic macules (≥3, at least 5-mm diameter)
2. Angiofibromas (≥3) or fibrous cephalic plaque
3. Ungual fibromas (≥2)
4. Shagreen patch
5. Multiple retinal hamartomas
6. Cortical dysplasias (cortical tubers and cerebral white matter radial migration lines)
7. Subependymal nodules
8. Subependymal giant cell astrocytoma
9. Cardiac rhabdomyoma
10. Lymphangioleiomyomatosis (LAM)
11. Angiomyolipomas (≥2)
Minor features
1. “Confetti” skin lesions
2. Dental enamel pits (>3)
3. Intraoral fibromas (≥2)
4. Retinal achromic patch
5. Multiple renal cysts
6. Nonrenal hamartomas
7. Sclerotic bone lesions
Definite diagnosis: Two major features or 1 major feature with ≥ 2 minor features, or detection of pathogenic TSC genes mutation
Possible diagnosis: Either 1 major feature or ≥ 2 minor features

TSC = tuberous sclerosis complex.

## 4. Conclusion

The patients described had TSC using clinical diagnostic criteria. Under the clinical diagnostic criteria of TSC, 4 of eleven major criteria and 3 of 7 minor criteria are skin features. Hence, awareness on skin features as clinical markers to suspect TSC should be emphasized in resource-limited countries.

## Acknowledgments

We are grateful to the medical personnel who were caring for the patients.

## Author contributions

**Conceptualization:** Belete Sisay, Abilo Tadesse.

**Data curation:** Belete Sisay, Abilo Tadesse, Abebe Gelaw, Desalew Getahun, Biruk Mulat, Weynishet Kebede, Yonathan Gebrewold.

**Investigation:** Belete Sisay, Abebe Gelaw, Weynishet Kebede, Yonathan Gebrewold.

**Validation:** Abilo Tadesse.

**Writing – original draft:** Belete Sisay, Abilo Tadesse.

**Writing – review & editing:** Belete Sisay, Abilo Tadesse, Abebe Gelaw, Desalew Getahun, Biruk Mulat, Weynishet Kebede, Yonathan Gebrewold.
